# A85 PATTERN OF SEMAGLUTIDE PRESCRIPTION IN A REAL-WORLD PATIENT COHORT

**DOI:** 10.1093/jcag/gwae059.085

**Published:** 2025-02-10

**Authors:** A Farahvash, M Lee, R Jain, L Jaakkimainen

**Affiliations:** University of Toronto Temerty Faculty of Medicine, Toronto, ON, Canada; University of Toronto Temerty Faculty of Medicine, Toronto, ON, Canada; University of Toronto Temerty Faculty of Medicine, Toronto, ON, Canada; University of Toronto Temerty Faculty of Medicine, Toronto, ON, Canada

## Abstract

**Background:**

The weight-loss effects of semaglutide has been studies in many randomized controlled trials. However, real-world data on its use is limited, particularly in the Canadian healthcare setting.

**Aims:**

This study aims to explore the pattern of semaglutide prescription and associated outcomes in an academic family medicine practice in Canada.

**Methods:**

We conducted a retrospective study of patients at the Sunnybrook Hospital family health team in Toronto, Ontario. Patients aged ≥18 years who were prescribed semaglutide between January 2018 and April 2024 were included. Baseline demographics and follow-up safety and efficacy measures were collected up to 16 months after semaglutide initiation.

**Results:**

Of the 9930 rostered patients, 368 (3.71%) were prescribed semaglutide during the study period. The average age of patients who were prescribed semaglutide was 57.7±14.1 years and 63.3% were female. The average BMI was 36.6±7.84 kg/m^2^ and 189 patients (51.4%) had diabetes. The indication for semaglutide prescription was weight-loss for 206 patients (56.0%), diabetes for 118 patients (32.1%), and both weight-loss and diabetes for 39 patients (10.6%). We observed that since 2018, there has been a growing number of family physicians prescribing semaglutide compared to endocrinologists. In 2023 and 2024, over 80% of all semaglutide prescribers for our patients were family physicians, and endocrinologists were in the minority. Despite being prescribed semaglutide, 20 patients (5.4%) did not initiate the medication and 66 (17.9%) discontinued it within 16 months. Reasons for discontinuation included gastrointestinal adverse effects (26/66, 39.4%), difficulty in access (30/66, 54.5%), and perceived lack of response (6/66, 9.1%).

Baseline and follow up weights were recorded for 211 patients (57.3%). Mean weight-loss was 7.36%. Mean weight-loss by 16 months was significantly greater in patients without diabetes (8.83% vs. 6.18%, p=0.003) and in females (8.30% vs. 5.90%, p=0.009), although there were more females without diabetes compared to males. The mean HbA1c at follow-up was 6.5±1.1%, which represents a significant reduction of 10.7% from baseline (p <0.001).

**Conclusions:**

Our analysis of a real-world cohort of patients who used semaglutide demonstrated its wight-loss effects, particularly in those without diabetes. Understanding the demographics of patients using semaglutide and its potential effects will help inform future prescription practices.

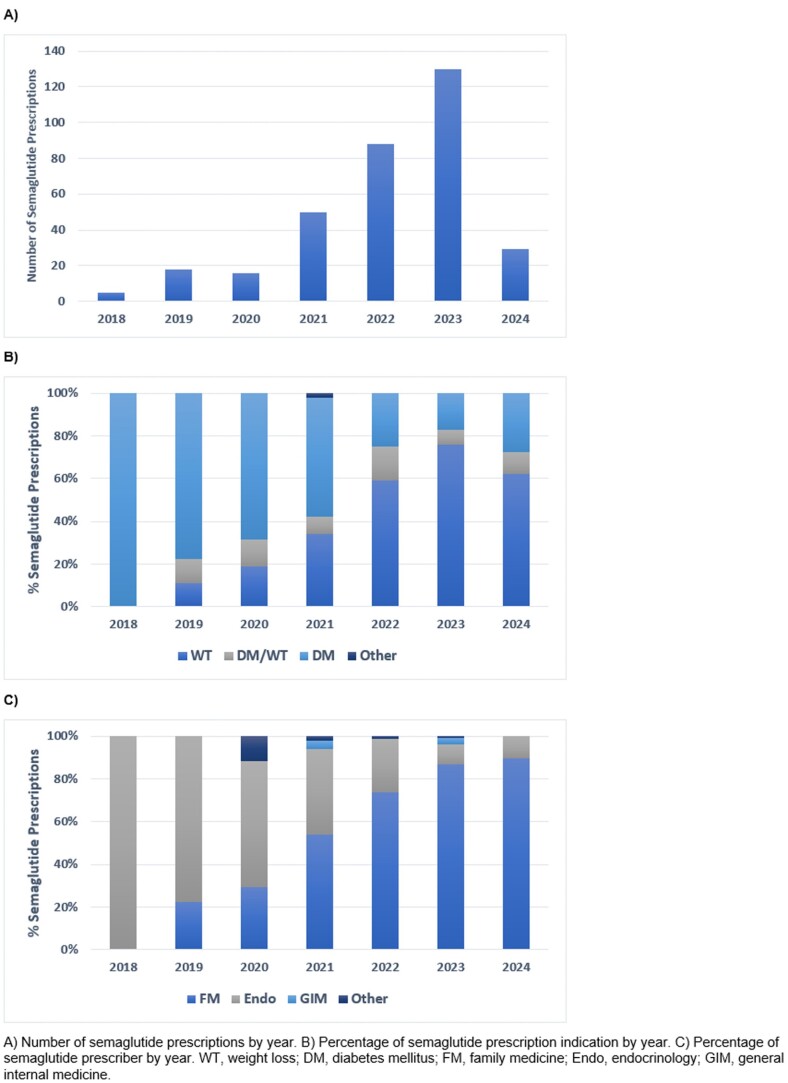

**Funding Agencies:**

None

